# The Role and Regulatory Mechanism of Hippo Signaling Components in the Neuronal System

**DOI:** 10.3389/fimmu.2020.00281

**Published:** 2020-02-19

**Authors:** Jinbo Cheng, Shukun Wang, Yuan Dong, Zengqiang Yuan

**Affiliations:** ^1^Center on Translational Neuroscience, College of Life and Environmental Science, Minzu University of China, Beijing, China; ^2^The Brain Science Center, Beijing Institute of Basic Medical Sciences, Beijing, China; ^3^Department of Biochemistry, Medical College, Qingdao University, Qingdao, China

**Keywords:** Hippo signaling, oxidative stress, neuronal system, neuroinflammation, diseases

## Abstract

The Hippo signaling pathway, an evolutionarily conserved protein kinase cascade, plays a critical role in controlling organ size, cancer development, and tissue regeneration. Recently, mounting evidence has suggested that Hippo signaling also has an important role in regulating immunity, including innate and adaptive immune activation. In the neuronal system, Our laboratory results, together with those from other studies, demonstrate that the Hippo signaling pathway is involved in neuroinflammation, neuronal cell differentiation, and neuronal death. In the present review, we summarize the recent findings pertaining to the function and regulatory mechanism of Hippo signaling components in the neuronal system, implicating the potential of Hippo signaling as a therapeutic target for the treatment of neuronal system diseases.

## Introduction

**Figure 1:**
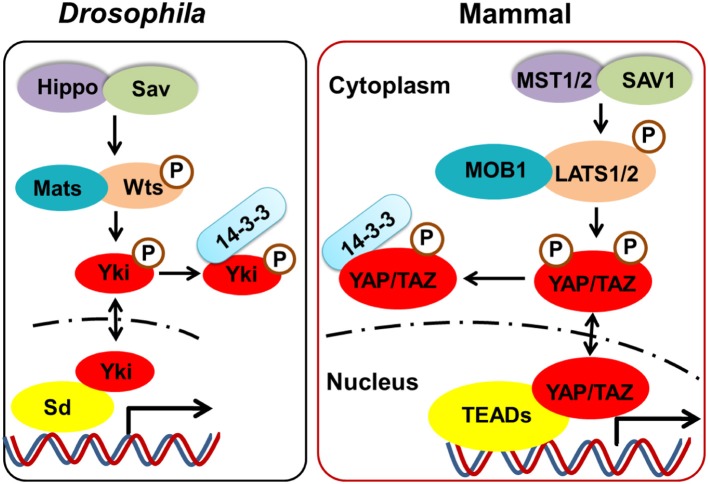
The core components of the Hippo pathway in *Drosophila* and mammalian. The core components of Hippo signaling in mammalian cells include MST1/2 (orthologs of Hippo in *Drosophila*) and their adaptor protein SAV1 (orthologs of Salvador in *Drosophila*). The phosphorylated of MST1/2 activates LATS 1/2 (orthologs of Warts in *Drosophila*), which in turn phosphorylates the downstream YAP (ortholog of Yki in *Drosophila*) or TAZ, resulting in cytosolic retention by interacting with protein 14-3-3. Meanwhile, unphosphorylated YAP or TAZ which is relatively enriched in the nucleus, bind to their key transcriptional factors TEADs (orthologs of Sd in *Drosophila*), thus regulating the cell proliferation, differentiation, and survival.

The Hippo signaling pathway, originally identified in *Drosophila*, plays a critical role in regulating cell contact inhibition, proliferation, differentiation, and apoptosis. As such, this pathway is closely associated with the control of organ size, cancer development, and autoimmune diseases (1–6). Importantly, the canonical Hippo signaling pathway is a highly-conserved evolutionary pathway. As shown in Figure 1, the core components of this pathway in mammalian cells include mammalian Ste20-like kinases 1/2 (MST1/2 [orthologs of Hippo in *Drosophila*]) and their adaptor protein, Sav family WW domain containing protein 1 (SAV1 [orthologs of Salvador in *Drosophila*]). The phosphorylation of MST1/2 activates large tumor suppressor 1/2 [LATS 1/2 (orthologs of Warts in *Drosophila*)], a downstream protein, which in turn phosphorylates the downstream Yes-associated protein (YAP [ortholog of Yki in *Drosophila*]) or a transcriptional coactivator with PDZ-binding motif (TAZ); this results in the prevention of nuclear translocation by interacting with cytosolic protein 14-3-3. Meanwhile, unphosphorylated YAP or TAZ are relatively enriched in the nucleus and bind to their key transcriptional factors, the TEA domain transcription factor (TEAD) family [TEAD 1–4 (orthologs of Sd in *Drosophila*)]; thus regulating the expression of many genes that enhance cell proliferation, differentiation, and survival.

Although discovered in *Drosophila*, the Hippo signaling pathway in mammals also plays critical role in the control of organ size, cancer development, and tissue regeneration. Recently, accumulating evidence has suggested that Hippo signaling is important in regulating cancer immunity, and for innate and adaptive immunity (7–13). However, compared with the peripheral immune system, the role and regulatory mechanism of this pathway in the neuronal system is less well-known. In the present review, we summarize the functions and regulation of the Hippo signaling pathway in the neuronal system, to update our understanding of this pathway and to raise awareness on its implications for drug development and the clinical treatment of disease.

## Expression of the Hippo Signaling Pathway in the Neuronal System

MST1 and MST2 are core components of the Hippo signaling pathway, that are highly expressed in several organs in mice. Double knockout (KO) of *MST1* and *MST2* results in early embryonic death (4); however, conventional deletion of *MST1* or *MST2* would only cause a failure of induction of tissue overgrowth or tumor development, thereby suggesting a functional redundancy in MST1 and MST2 (7). Consistent with this observation, organ overgrowth has been reported in many tissue-specific double KOs of *MST1* and *MST2*, such as in the liver, intestine and heart (3–5, 14), suggesting that mammalian MST1 and MST2 are important in the regulation of development and growth.

Recently, mounting evidence has shown that the Hippo signaling pathway also plays a critical role in the neuronal system. Using an RNA-Seq transcriptome and splicing database of glia, neurons, and vascular cells of the cerebral cortex (15), we analyzed the expression levels of the core components of the Hippo signaling pathway. As shown in Table 1, MST1 and MST2 are highly expressed in most cell types in the brain, including astrocytes, neurons, oligodendrocyte progenitor cells (OPCs), newly formed oligodendrocytes, myelinating oligodendrocytes, microglia, and endothelial cells. The adaptor protein SAV1 also has a similar expression pattern, and downstream LATS1 and LATS2 are also highly expressed in all of these cell types. However, the co-transcription factor YAP is highly expressed only in astrocytes and endothelial cells, with low expression in neurons, OPCs, newly formed oligodendrocytes, myelinating oligodendrocytes, and microglia. Conversely, TAZ exhibits a different expression pattern compared with YAP, and it is highly expressed in all of these cell types, suggesting that YAP and TAZ may have different roles in the diverse cell types of the neuronal system. Different expression patterns have also been observed in TEAD 1, 2, 3, and 4. Specifically, TEAD1 is highly expressed in astrocytes and neurons, but relatively less expressed in OPCs and endothelial cells. TEAD2 is highly expressed in astrocytes and endothelial cells, while TEAD3 is expressed in astrocytes and neurons, with relatively lower expression levels in OPCs and microglia. TEAD4 on the other hand only has relatively high expression levels in endothelial cells. Hence, these diverse expression patterns of components of the Hippo signaling pathway suggest their diverse roles among the different cell types in the brain.

**Table 1:** The expressions of Hippo components in the brain.

**Gene name**	**Cell type in the brain**						
	**Astrocyte**	**Neuron**	**OPC**	**Newly formed oligodendrocyte**	**Myelinating oligodendrocyte**	**Microglia**	**Endothelial**
*MST1(STK4)*	+^***^	+^**^	+^***^	+^***^	+^**^	+^***^	+^**^
*MST2(STK3)*	+^**^	+^**^	+^**^	+^**^	+^**^	+^**^	+^***^
*SAV1*	+^***^	+^**^	+^***^	+^**^	+^**^	+^***^	+^***^
*LATS1*	+^***^	+^***^	+^***^	+^***^	+^**^	+^**^	+^***^
*LATS2*	+^***^	+^**^	+^***^	+^***^	+^**^	+^***^	+^***^
*YAP*	+^***^	+^*^	+^*^	+	+	+	+^***^
*TZA*	+^***^	+^***^	+^***^	+^***^	+^***^	+^***^	+^***^
*TEAD1*	+^***^	+^***^	+^**^	+	+	+	+^**^
*TEAD2*	+^***^	+	+	-	-	+	+^***^
*TEAD3*	+^***^	+^***^	+^**^	+	+	+^**^	+
*TEAD4*	+	+	+	+	+	+	+^**^

An expression level of 0.5 < FPKM ≤ 1.0 was indicated as

“*”; 1.0 < FPKM ≤ 5.0 was indicated as

“**”; FPKM > 5.0 was indicated as

“***” *(FPKM, fragments per kilobase of transcript sequence per million mapped fragments)*.

## Role of Hippo Signaling in Neural Stem Cells

In the vertebrate brain, neural stem cells (NSCs) are self-renewing, multipotent cells that generate neurons and glial cells during embryonic development (16). It is noteworthy that some NSCs persisting in the subgranular and subventricular zones continue to produce neurons throughout life. Consequently, different states of NSCs exist and are tightly regulated in the brain. Usually, NSCs either undergo symmetrical or asymmetrical cell division into two daughter cells. In symmetrical cell division, both daughter cells are stem cells; however, in asymmetric division, NSCs produce differentiated daughter cells and stemness daughter cells (17). There are also some inactive state NSCs or quiescent NSCs, when proliferation is not required (18).

Recently, mounting evidence has shown that the Hippo pathway plays an important role in regulating NSC physiology. In neural progenitors, inactivation of LATS1/2 kinases (upstream inhibitors of YAP/TAZ) cause massive apoptosis through the induction of YAP/TAZ activation, and upregulating a series of genes associated with cell growth and proliferation (19). Additionally, overexpression of YAP/TAZ in the mouse embryonic brain induced cell localization in the ventricular zone by increasing stemness. Moreover, introduction of YAP/TAZ increased the frequency and size of neurospheres in a TEAD-dependent manner, as a TEAD binding-defective YAP mutant failed to induce this phenotype (20). The results from our study demonstrated that bone treatment with morphogenetic protein-2 (BMP2) could inhibit the proliferation of embryonic NSCs; meanwhile, under the condition of YAP knockdown, BMP2 does not further reduce neurosphere formation, suggesting the presence of cross-talk between BMP2 signaling and the Hippo-YAP pathway. Mechanically, under BMP2 stimulation, Smad1/4 complex is transported into the nucleus, where it competes with TEAD1 for binding to YAP, resulting in the inhibition of its transcriptional activity. Furthermore, under the condition of cyclin D1 (ccnd1) knockdown, an important downstream target gene of YAP-TEAD signaling, BMP2 fails to show additional inhibitory effect on mouse NSC proliferation (21). YAP is also involved in neocortical astrocytic differentiation and proliferation during brain development in mice. Conditional KO of *YAP*, using Nestin-cre or GFAP-cre, decreases the number of neocortical astrocytes and impairs astrocytic proliferation through the BMP2-YAP-SMAD1 pathway (22). Furthermore, the loss of Hippo or Warts induces the growth and proliferation of NSCs in the *Drosophila* nervous system, suggesting that Hippo signaling also plays a critical role in maintaining NSC quiescence (23).

## Role and Mechanism of Hippo Signaling in Neuronal Cell Death

It has been established that Hippo signaling is involved in the control of organ size and tumor development. Hippo inhibition results in higher activity of YAP and leads to tumorigenesis; however, its activation plays a role in neurodegeneration by mediating oxidative stress-induced neuronal death. Oxidative stress activates MST1 and then induces either YAP-dependent or YAP-independent cell proliferation and cell death (24, 25).

The mammalian fork-head transcription factors of the O class (FOXOs) are well-characterized substrates of MST1. By stimulating oxidative stress, we found that MST1 phosphorylates FOXO proteins, disrupts their interaction with protein 14-3-3, and promotes FOXO nuclear translocation, thereby inducing cell death in neurons (26, 27). Apart from phosphorylation, we also found that methylation of FOXO3 was involved in neuronal cell death. Consequently, Methyltransferase Set9 methylates FOXO3 at lysine 270, leading to the inhibition of Bim expression and neuronal apoptosis (28). Moreover, we discovered that the upstream kinase c-ABL, a non-receptor tyrosine kinase involved in the oxidative stress-induced neuronal cell death (29, 30), phosphorylates MST1 at Y433, which triggers the stabilization and activation of MST1, and increases the interaction between MST1 and FOXO3, thereby leading to neuronal cell death (31). Finally, we discovered that histone deacetylase 2 (HDAC2) could form a complex with FOXO3a and regulate FOXO3a-dependent gene transcription and oxidative stress-induced neuronal cell death, which describes a novel, epigenetic modification-dependent regulatory mechanism of FOXO3a-mediated selective gene transcription (32).

**Figure 2:**
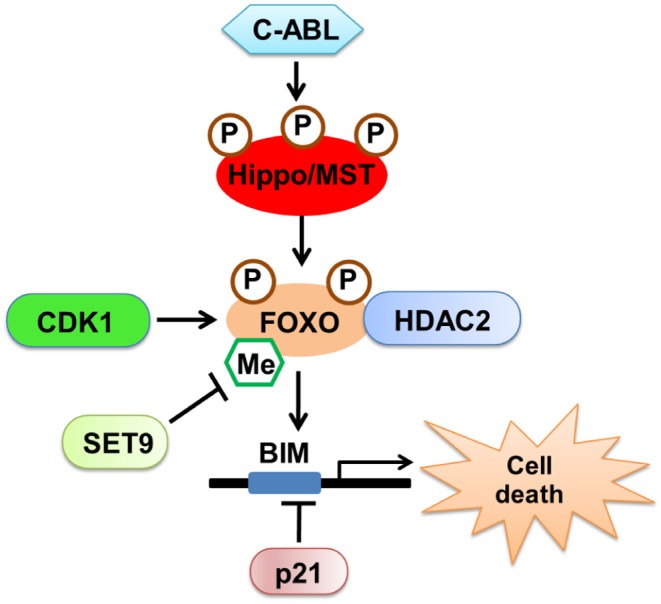
The regulatory mechanism of Hippo/MST in neuronal cell death. Oxidative stress activates upstream kinase c-ABL, which phosphorylates MST1 and triggers the stabilization and activation of MST1. MST1 could phosphorylate FOXO proteins, promote FOXO nuclear translocation and induce cell death in neurons. Additionally, CDK1 and SET9 could regulate the modification of FOXO proteins. HDAC2 could form a complex with FOXO3a and regulate FOXO3a-dependent gene transcription and oxidative stress-induced neuronal cell death.

Interestingly, there is a functional interaction between Hippo-YAP signaling and FOXO1 in treatments that induce oxidative stress. YAP acts as a nuclear co-factor of FOXO1, which modulates the FOXO1-mediated antioxidant response. Activation of Hippo antagonizes YAP-FOXO1, leading to increased ischemia/reperfusion (I/R)-induced cell death through downregulation of catalase and MnSOD (33). These results revealed that MST1 could induce both YAP-dependent and YAP-independent gene transcription on oxidative stress, which both determine cell survival or death in the neuronal system (Figure 2).

## Role of Hippo Signaling in Neuronal System Diseases

Accumulating evidence has shown that dysfunctions in Hippo signaling are involved in multiple neuronal system diseases. As shown in Table 2, in *MST1* and *MST2* KO mice, MST2—but not MST1—was shown to be a critical regulator of caspase-mediated photoreceptor cell death in a mouse model of retinal detachment (RD). Mechanically, KO of *MST2* decreases caspase-mediated photoreceptor cell death and proinflammatory cytokines, such as monocyte chemoattractant protein 1 and interleukin (IL)-6 during the early phase of RD (36). Moreover, MST1 has been reported to function as a key determinant of neurodegeneration in amyotrophic lateral sclerosis (ALS) (35). Furthermore, KO of *MST1* delays disease onset and extends survival in mice expressing the human SOD1 G93A mutant. Mechanically, deficiency of *MST1* also decreases the activation of p38 mitogen-activated protein kinase and caspases, and impairs autophagy in spinal cord motor neurons. Consistently, in *Drosophila*, Warts signaling is required for autophagic flux in neurons, and mutants of the Warts pathway cause progressive polyglutamine (PolyQ)-mediated neurodegeneration in the adult stage. Importantly, phosphorylated MST1—the active form of MST1—was reported to be significantly increased in the post-mortem cortex of patients with Huntington's disease (HD). Meanwhile, YAP nuclear localization was decreased in both HD post-mortem cortex and neuronal stem cells derived from HD patients (24), suggesting that the activation of Hippo signaling may contribute to HD.

**Table 2:** The functions of Hippo components in the neuronal system diseases.

**Gene**	**Diseases**	**Function/change**	**References**
*Mst1*	ICH	Decrease neuronal cell death and inflammatory reaction, leading to the reduced brain edema, blood-brain barrier damage, and neurobehavioral impairment	(34)
	ALS	Decreased the activation of p38 mitogen-activated protein kinase and caspases, impaired the autophagy in spinal cord motor neurons	(35)
	HD	Phosphorylated MST1 increased in post-mortem HD cortex	(24)
*Mst2*	RD	A critical regulator of caspase-mediated photoreceptor cell death	(36)
*Wts*	Aging	Causes progressive polyglutamine (PolyQ)-mediated neurodegeneration in the adult stage	(24)
*Yap*	HD	Decreased in the both HD post-mortem cortex and neuronal stem cells	(24)
	I/R	Decreased cerebral edema, smaller brain infarct sizes, and improved neurologic function	(37)

*ICH, intracerebral hemorrhage; ALS, amyotrophic lateral sclerosis; HD, Huntington's disease; RD, retinal detachment; I/R, ischemia/reperfusion*.

Additionally, MST1 was reported to be activated in a model of intracerebral hemorrhage established by injecting autologous blood into the right basal ganglia. Hence, genetic knockdown *MST1* or chemical inhibition could effectively reduce the levels of p-LATS1 and p-YAP, and decrease neuronal cell death and inflammatory reactions, leading to a reduction in brain edema, blood-brain-barrier (BBB) damage, and neurobehavioral impairment (34). Furthermore, it has been reported that I/R resulted in decreased levels of YAP and TAZ; hence, the intraperitoneal injection of the YAP agonist, dexamethasone, led to decreased BBB permeability, decreased cerebral edema, smaller brain infarct sizes, and improved neurological function, suggesting a neuroprotective effect of YAP on the I/R-induced damaged brain (37). Moreover, supplementation with melatonin could activate the YAP-Hippo pathway; thus enhancing mitochondrial fusion and ultimately reducing brain reperfusion stress. Mechanically, the YAP-Hippo pathway regulates melatonin-modified OPA1 expression, while blockade of the YAP-Hippo pathway results in neuronal cell death and mitochondrial damage (38). Additionally, the administration of biodegradable selenium (Se) nanoparticles led to the protection of axons in the hippocampus region and myelination of the hippocampal area after cerebral ischemic stroke. Mechanically, Se administration suppressed excessive inflammation and oxidative metabolism, and Hippo signaling was shown to be involved in this process (39).

## Role of Hippo Signaling in Neuroinflammation

Apart from the important role of Hippo signaling in controlling organ size and cancer development, its role in immunity activation has recently been extensively studied. The key component of the Hippo signaling pathway, MST1, is highly expressed in lymphoid tissues. *MST1* KO mice also exhibit normal T cell development, but low numbers of mature naive T cells and relatively normal numbers of effector/memory T cells (7). In 2012, Abdollahpour et al. reported a homozygous premature termination mutation of MST1 with a novel clinical phenotype including T- and B-cell lymphopenia, intermittent neutropenia, and atrial septal defects; this suggest that *MST1* deficiency is a novel human primary immunodeficiency syndrome. Moreover, enhanced loss of mitochondrial membrane potential and increased susceptibility to apoptosis was observed in *MST1*-deficient lymphocytes and neutrophils (8). In the same year, Nehme et al. reported a similar primary immunodeficiency phenotype associated with *MST1* deficiency that was characterized by a progressive loss of naive T cells, recurrent bacterial and viral infections, and autoimmune manifestations (10). Subsequently, in 2015, Halacli et al. reported a novel STK4 mutation with clinical features including autoimmune cytopenias, viral skin and bacterial infections, mild onychomycosis, mild atopic, and seborrheic dermatitis, lymphopenia, and intermittent mild neutropenia (40); these features are similar with those of *DOCK-8* deficiency, a form of autosomal recessive (AR) hyperimmunoglobulin E syndrome. Hence, these results strongly indicate that MST1 plays a critical role in the immune system.

The diverse KO of major components of Hippo signaling models and some functional studies have also revealed that Hippo signaling plays an essential role in both innate and adaptive immunity. In innate immunity, the loss of Hippo or activation of Yki in fat bodies (the *Drosophila* immune organ) results in a decreased antimicrobial response and increase vulnerability to infection by Gram-positive bacteria. Mechanically, Gram-positive bacteria could activate Hippo-Yki signaling through Toll-Myd88 signaling, in which Yki directly regulates the transcriptional activity of Cactus, the *Drosophila* IκB homolog (11). However, an opposite role of Hippo signaling was shown in mammalian macrophages. Furthermore, *MST1/2* deficient bone-marrow-derived macrophages exhibited higher toll-like receptor 4-mediated nuclear factor (NF)-κB activation, resulting in increased levels of some pro-inflammatory cytokines, such as IL-6, tumor necrosis factor-alpha, and IL-1β (41). Moreover, the downstream effector YAP was reported to negatively regulate antiviral immune response. Deficiency of *YA*P also resulted in enhanced innate immunity, and a decreased viral load, and morbidity *in vivo* (42). Additionally, YAP functions as a transcriptional coactivator of β-catenin in mesenchymal stem cell-mediated immune regulation. Deficiency of macrophage YAP or β-catenin increased XBP1-mediated NLRP3 expression, thus regulating macrophage polarization. In adaptive immunity, MST1 and MST2 have been demonstrated to also be important in T- and B-cell development, differentiation, and function (13, 43–45).

The functions of Hippo signaling in the neuronal system have recently been elucidated. We found that KO of *MST1* in microglia protects from acute cerebral I/R-induced neuroinflammation and brain injury. Mechanically, in the acute cerebral I/R condition, MST1 directly phosphorylates IκB at residues S32 and S36, thus regulating the activation of NF-κB signaling in microglia. Deficiency of *MST1* in microglia significantly suppressed NF-κB signaling and microglial activation. Moreover, we found that Src kinase functions upstream of MST1-IκB signaling, and that administration of the Src inhibitor AZD0530 exhibited a phenotype similar to *MST1* deficiency in microglia (46). Consistent with this result, suppression of MST1 was also reported to reduce early brain injury after subarachnoid hemorrhage in mice by inhibiting NF-κB/MMP-9 signaling (47). These results suggest that MST1 positively regulates NF-κB signaling and that inhibition of MST1 plays a protective role in microglial activation-induced neuroinflammation. Moreover, YAP has been reported to be highly expressed in astrocytes, and *YAP* deletion induced the over-activation of astrocytes, along with microglial activation and BBB dysfunction in mice (48). Mechanically, KO of *YAP* in astrocytes increased the action of the JAK-STAT inflammatory pathway; thus, inducing reactive astrogliosis. Results from our laboratory demonstrated that *YAP* conditional KO (cKO) in the lens led to cataracts in mice (49). Mechanistically, *YAP* cKO reduced proliferation of epithelial cells, delayed fiber cell denucleation, and increased cellular senescence in the lens; the inflammation levels were also significantly altered in *YAP* cKO mice. Collectively, these results suggest that Hippo signaling is also important in neuroinflammation in the neuronal system, which may not be consistent with its functions in the peripheral immune system; thus, warranting further investigations.

## Concluding Remarks

The Hippo signaling pathway is not only critical in controlling organ size, cancer development and tissue regeneration, but it is also important in regulating immunity, including the activation of the innate and adaptive immune systems. Recently, multiple studies have shown that Hippo signaling components paly critical role in the neuronal system, including the regulation neural stem cell proliferation and differentiation, oxidative stress-induced neuronal cell death, and in neuroinflammation; thus implicating a potential therapeutic target for the treatment of neuronal system diseases. However, the role and the regulatory mechanism of Hippo signaling in the neuronal system still requires clarification, especially in different diseases or environmental conditions.

## Author Contributions

JC wrote the manuscript and prepared the figure. SW and YD review the manuscript. JC and ZY conceived the review topic and performed a comprehensive review of the literature.

### Conflict of Interest

The authors declare that the research was conducted in the absence of any commercial or financial relationships that could be construed as a potential conflict of interest.
